# Altered glial marker expression in autistic post-mortem prefrontal cortex and cerebellum

**DOI:** 10.1186/2040-2392-5-3

**Published:** 2014-01-10

**Authors:** Catherine Edmonson, Mark N Ziats, Owen M Rennert

**Affiliations:** 1 Laboratory of Clinical and Developmental Genomics, National Institute of Child Health and Human Development, National Institutes of Health, 49 Convent Drive, Building 49, Room 2C078, Bethesda, MD 20814, USA; 2 University of Florida College of Medicine, 1600 SW Archer Rd, Gainesville, FL 32603, USA; 3 University of Cambridge, Robinson College, Grange Rd, Cambridgeshire CB3 9AN, UK; 4 Baylor College of Medicine MSTP, One Baylor Plaza, Houston, TX 77030, USA

**Keywords:** Astrocyte, Autistic disorder, Gene expression, Glia, Interneuron, Microglia, Neuron

## Abstract

**Background:**

The cellular mechanism(s) underlying autism spectrum disorders (ASDs) are not completely understood, but ASDs are thought to ultimately result from disrupted synaptogenesis. However, studies have also shown that glial cell numbers and function are abnormal in post-mortem brain tissue from autistic patients. Direct assessment of glial cells in post-mortem human brain tissue is technically challenging, limiting glial research in human ASD studies. Therefore, we attempted to determine if glial cell-type specific markers may be altered in autistic brain tissue in a manner that is consistent with known cellular findings, such that they could serve as a proxy for glial cell numbers and/or activation patterns.

**Methods:**

We assessed the relative expression of five glial-specific markers and two neuron-specific markers via qRT-PCR. We studied tissue samples from the prefrontal cortex (PFC) and cerebellum of nine post-mortem autistic brain samples and nine neurologically-normal controls. Relative fold-change in gene expression was determined using the ΔΔC_t_ method normalized to housekeeping gene β-actin, with a two-tailed Student’s *t*-test *P* <0.05 between groups considered as significant.

**Results:**

Both astrocyte- and microglial-specific markers were significantly more highly expressed in autistic PFC as compared to matched controls, while in the cerebellum only astrocyte markers were elevated in autistic samples. In contrast, neuron-specific markers showed significantly lower expression in both the PFC and cerebellum of autistic patients as compared to controls.

**Conclusions:**

These results are in line with previous findings showing increased glial cell numbers and up-regulation of glial cell gene expression in autistic post-mortem brain tissue, particularly in the PFC, as well as decreased number of neurons in both the PFC and cerebellum of autistic patients. The concordance of these results with cell-level studies in post-mortem autistic brain tissue suggests that expression of glial cell-type specific markers may serve as a useful alternative to traditional cellular characterization methods, especially when appropriately-preserved post-mortem tissue is lacking. Additionally, these results demonstrate abnormal glial-specific gene expression in autistic brains, supporting previous studies that have observed altered glial cell numbers or activation patterns in ASDs. Future work should directly assess the correlation between cell-type specific marker levels and cell number and activation patterns.

## Background

Autism spectrum disorders (ASDs) are neurodevelopmental syndromes defined by impairments in language, verbal and non-verbal communication, and restrictive/repetitive patterns of behavior [1]. ASD symptoms manifest within the first two years of life, with their severity and presentation varying considerably between individuals, thus yielding the 'spectrum’ classification. ASDs are estimated to affect 1 in 88 children in the United States, and the prevalence of ASDs is at least four times more common in males than females [2].

The etiology of autism is complex and the neurobiological mechanism(s) that result in the clinical phenotype remain to be fully understood. However, there is strong evidence that the autism phenotype ultimately results from aberrant synaptic wiring in the developing brain [3]. In particular, studies have shown that long-distance communication between disparate neocortical areas may be disrupted in ASDs, causing delays in information processing within the brain that manifest as the communication, language, and social development problems seen in children with autism [4]. Additionally, parallel research has shown that neuronal micro-circuitry within brain areas may also be disrupted in ASDs, and that this may result in local processing deficits within brain regions related to higher functioning, such as the prefrontal cortex (PFC) [5]. Underlying these circuit disruptions is a large body of evidence that has demonstrated decreased numbers of neurons (and their various subtypes) throughout the autistic brain by early childhood in post-mortem studies [6].

In addition to the body of evidence implicating aberrant local and long-distance synaptic dysfunction in ASDs, many studies have demonstrated microglial and astrocyte dysfunction in ASD brains. For instance, post-mortem pathological studies of autistic brain using immunocytochemistry (IHC) and/or stereology have identified microglial activation patterns [7-9], and have demonstrated increased microglial cell density in multiple brain regions [8,10]. Glial activation refers to the well characterized cascade of events that occurs upon reaction of glia to stimuli, resulting in their mobilization, release of cytokines, and ability to phagocytize, among other processes [11]; glial activation is associated with specific gene expression patterns that are distinct from 'resting’ glial gene expression [12]. Furthermore, positron emission tomography (PET) using a microglial-specific radiotracer also demonstrated microglial activation in multiple brain regions of autistic cases [13]. Additionally, studies in a Rett syndrome mouse model, a single-gene deletion disorder with autism as a component, have also demonstrated cellular microglial abnormalities [14], and a remarkable study demonstrated that autistic-like phenotypes can be partially reversed by replacing mutant Mecp2 (-/-) microglia with their respective wild-type cells [15].

Increased numbers of astrocytes, with altered cell size and branching patterns, have also been demonstrated in post-mortem autistic brains [16]. Additionally, astrocyte-specific cell marker proteins are increased in multiple autistic brain regions [17,18]. Similar to microglial studies in ASD mouse models, astrocytes have been shown to be abnormal in a number of single-gene ASD models, including Rett [19,20], Fragile X [21], and Tuberous Sclerosis [22]. In parallel to the aforementioned microglial study, it was also shown that replacing mutant astrocytes in Mecp2 (-/-) mice can correct some aspects of the phenotype [23].

However, it is not clear how these separate lines of evidence—one demonstrating immune/glial dysfunction in ASDs and the other implicating synaptic abnormalities—may converge into a common mechanism in the autistic brain that ultimately results in the shared clinical phenotype. Because separate studies have shown that microglia and astrocytes play critical roles in sculpting developing synapses during normal neurodevelopment [24,25], it is reasonable to hypothesize that inherent defects or aberrant numbers of microglia and astrocytes in the developing autistic brain may be causative of the synaptic abnormalities by affecting the proper wiring of developing neuronal connections. However, because appropriately-preserved post-mortem autistic brain tissue is lacking [26], cellular-level studies assessing glial numbers and activation in human autistic brains have been limited. Moreover, quantification of cell numbers in postmortem tissue by stereology is technically challenging, further limiting the ability of researchers to assess the few appropriately-preserved tissue samples that are available. Finally, no studies have concurrently specifically assessed for microglia, astrocytes, and neurons in the same set of autistic brain samples. As a consequence, a comprehensive understanding of the relationship between glial and neuron cells in autistic brains is needed.

Therefore, the purpose of this study was two-fold. First, we sought to determine if microglia, astrocyte, and neuron-specific markers were altered in post-mortem autistic brain tissue, in order to further investigate the role of glia in ASDs. Then, we determined if glial and neuronal cell-type specific marker expression patterns are consistent with known cellular-level findings, because gene expression studies of post-mortem human brain are often easier to perform than cell-level studies, and therefore this approach may serve as a valuable 'screening’ assay to infer relative cell proportions.

In this study, we compared internally-normalized mRNA expression levels of microglial, astrocyte, and neuronal cell-type specific marker genes in post-mortem brain tissue from patients with autism and healthy controls. Our results provide further evidence for a role of glia in autism pathology, and suggest that assessment of glial cell-type specific markers may serve as a proxy for relative cellular numbers or activation patterns.

## Methods

### Post-mortem brain samples

Post-mortem brain tissue was obtained from the National Institute of Child Health and Human Development Brain and Tissue Bank, MD, USA. This source obtained consent to use brain tissue for research from each patient or their guardian prior to his/her death, and their protocol was approved by their Institutional Review Board. No patient-specific identifiable information was obtained. Because multiple brain regions have been implicated in ASDs, we performed our analysis in two separate areas that have been consistently demonstrated as abnormal in autism—the PFC and the cerebellum. We obtained post-mortem PFC brain tissue from five individuals with autism and from five healthy controls (Table 1). We obtained post-mortem cerebellum brain tissue from four individuals with autism and four healthy controls. The majority of sample pairs (PFC and cerebellum) were derived from the same donor brain. All cases were Caucasian males, and case controls were matched by age as closely as possible.

**Table 1:** Clinical characteristics and RNA quality of autistic and control brain samples

**Sample#**	**UMB #**	**Diagnosis**	**Brain area**	**Age (yr)**	**Cause of death**	**PMI (h)**	**RNA quality**		
							**A260/280**	**A260/230**	**RIN**
1	5308*	Autism	PFC	4.5	Skull fracture	21	2.051	2.266	4.9
2	1349	Autism	PFC	5.6	Drowning	39	2.044	2.232	4.3
3	5144	Autism	PFC	7.2	Rhabdomyosarcoma	3	2.058	2.271	5.4
4	5302*	Autism	PFC	16.3	DKA	20	2.031	2.238	4
5	4999*	Autism	PFC	20.8	Cardiac arrhythmia	14	2.039	2.232	6
6	4670*	Control	PFC	4.6	Commotio Cordis	17	2.048	2.276	5.2
7	1185	Control	PFC	4.7	Drowning	17	2.026	2.243	4.7
8	4898	Control	PFC	7.7	Drowning	12	2.056	2.183	5.9
9	4848*	Control	PFC	16.7	Drowning	15	2.044	2.185	6.7
10	4727*	Control	PFC	20.5	Multiple injuries (MVA)	5	2.066	2.209	6.5
11	5308*	Autism	Cere	4.5	Skull fracture	21	2.087	1.781	7.3
12	4899	Autism	Cere	14.3	Drowning	9	2.077	2.314	9.3
13	5302*	Autism	Cere	16.3	DKA	20	2.083	1.646	2.2
14	4999*	Autism	Cere	20.8	Cardiac arrhythmia	14	2.081	2.114	9.2
15	4670*	Control	Cere	4.6	Commotio Cordis	17	2.088	2.161	6.1
16	4722	Control	Cere	14.5	Multiple injuries (ATV)	16	2.073	2.828	6.5
17	4848*	Control	Cere	16.7	Drowning	15	2.087	2.327	6.8
18	4727*	Control	Cere	20.5	Multiple Injuries (MVA)	5	2.067	2.307	7.2

PFC: Prefrontal cortex; Cere: Cerebellum; UMB: University of Maryland Brain Bank sample number; PMI: Post-mortem interval; DKA: Diabetic ketoacidosis; ATV: All-terrain vehicle; MVA: Motor vehicle accident; RIN: RNA integrity number. *Indicates both prefrontal cortex and cerebellum samples were assessed from the same donor brain.

### RNA isolation and quality control

RNA isolation and quality control analysis were performed as previously described [27]. Briefly, total RNA was extracted using TRIZOL Reagent (Life Technologies, Carlsbad, CA, USA) according to the manufacturer’s protocol. Quantification of RNA was performed using a NanoDrop ND-1000, and RNA integrity was assessed using an Agilent Bioanalyzer 2100 (Table 2).

**Table 2:** Primers used for qRT-PCR

**Primer name**	**Primer sequence (5′ to 3′)**	**OD**	**MW**	**% GC content**	**T**_**m **_**(°C)**
ActinB-F	AGAAAATCTGGCACCACACC	4.1	6064	50	60.4
ActinB-R	AGAGGCGTACAGGGATAGCA	4.3	6240.1	55	62.4
Trem2-F	CCGGCTGCTCATCTTACTCT	3.3	5995	55	62.4
Trem2-R	AGTCATAGGGGCAAGACACC	4.2	6160	55	62.4
Dap12-F	GAGACCGAGTCGCCTTATCA	3.8	6102	55	62.4
Dap12-R	GTCATGATTCGGGCTCATTT	3.7	6114.1	45	58.4
Cx3cr1-F	GCAGATCCAGAGGTTCCCTT	3.7	6093	55	62.4
Cx3cr1-R	TAACAGGCCTCAGCCAAATC	3.9	6055	50	60.4
Gfap-F	CTGCGGCTCGATCAACTCA	3.5	5748.8	57.9	62.3
Gfap-R	TCCAGCGACTCAATCTTCCTC	3.6	6277.1	52.4	62.7
Nefl-F	AGCTGGAGGACAAGCAGAAC	4.4	6209.1	55	62.4
Nefl-R	TGCCATTTCACTCTTTGTGG	3.5	6065	45	58.4
Parvalbumin-F	CTGGAGACAAAGATGGGGAC	4.3	6240.1	55	62.4
Parvalbumin-R	CAGAGAGGTGGAAGACCAGG	4.4	6265.1	60	64.5
Aif1-F	AGCAGTGATGAGGATCTGCC	4.0	6182.1	55	62.4
Aif1-R	AGCATTCGTTTCAGGGACAT	3.9	6132.1	45	58.4

F: Forward; R: Reverse; OD: Optical density; MW: Molecular weight; T_m_: Melting temperature.

### Reverse transcriptase reaction

Total RNA (1 μg) was used in a 20 μL reverse transcriptase reaction to synthesize cDNA with SuperScript3 Reverse Transcriptase (Life Technologies) according to the manufacturer’s protocol. Briefly, 1 μg of total RNA was added to an aqueous solution containing 250 ng/μL of random hexamer and 10 mM deoxyribonucleotide triphosphate. The RNA was denatured for 5 minutes at 65°C and then snap cooled on ice for 2 minutes. After which 0.1 M DTT, 5× First-Strand Buffer (250 mM Tris–HCl, 375 mM KCl, 15 mM MgCl_2_), RNaseOUT Recombinant Ribonuclease Inhibitor (40 Units/μL), and SuperScript3 Reverse Transcriptase (200 Units/μL) were added into each sample mixture. The reaction was carried out under the following conditions: 25°C for 5 minutes, 50°C for 60 minutes, and 70°C for 15 minutes. The cDNA produced from the reaction was diluted to 0.25× with nuclease free water.

### Real time quantitative PCR

SYBR Green Expression Assay System (Applied Biosystems, Foster City, CA, USA) was used to measure relative, normalized, mRNA expression levels. We assessed four separate microglial-specific cell surface genes: Triggering receptor expressed on myeloid cells 2 (*TREM2*), *DAP12*, CX3C chemokine receptor 1 (*CX3CR1*), and allograft inflammatory factor 1 (*AIF1*) [28]. Two cell type specific intermediate filaments, glial fibrillary acidic protein (*GFAP*), which is astrocyte-specific [29], and the pan-neuronal cell marker *NEFL*[30], were used to assess for astrocytes and neurons, respectively. Additionally, we assessed for GABAergic interneurons specifically with parvalbumin (*PVL*) [31]. The intermediate filament housekeeping gene beta-actin (*ACTB*) was used as a control. Forward and reverse primer sequences were generated using Primer3 software and synthesized by Eurofins MWG Operon (Huntsville, AL, USA) (Table 2).

Quantitative reverse transcriptase polymerase reaction (qRT-PCR) was performed using an ABI Prism 7900 Sequence Detection System (Life Technologies) with a 96-well format. Each qRT-PCR reaction contained 6.5 μL water, 12.5 μL SYBR Green master mix (Applied Biosystems), 1 μL forward primer (10 μM), 1 μL reverse primer (10 μM), and 4 μL of cDNA (0.25×). Data was collected using the SDS2.3 Program (Applied Biosystems) under the following run parameters: 48°C for 30 minutes, 95°C for 10 minutes, 40 cycles of 95°C for 15 seconds, 60°C for 1 minute, and a final dissociation stage.

### Data analysis

The target genes and the endogenous controls were measured with technical triplicates in each qRT-PCR reaction, and all genes were assessed in three separate, independent qRT-PCR runs. The cycle threshold number (C_t_) was calculated using RQ Manager 1.2 Software (Applied Biosystems). Relative expression of each target gene was normalized to *ACTB* using the ΔΔ C_t_ method. All *P* values reported are based on a two-tailed Student’s *t*-test. Only results with a *P* value less than 0.05 were considered significant.

## Results

The average post-mortem interval (PMI) was not significantly different between autistic and control tissue samples (Table 1; ASD = 17.9 h, ctrl = 13.2 h, *P* = 0.16). This remained true after sub-stratifying by brain region (ASD PFC = 19.4 h, ctrl PFC = 13.2 h, *P* = 0.28; and ASD cerebellum = 16.0 h, ctrl cerebellum = 13.25 h, *P* = 0.48). RNA isolated from post-mortem brain tissue was generally of high quality, and the RNA Integrity Number (RIN) was not significantly different between autism and controls (ASD = 5.84, ctrl = 6.18, *P* = 0.67). The RIN was also not significantly different after sub-stratifying by brain region (ASD PFC = 4.92, ctrl PFC = 5.80, *P* = 0.13; and ASD cerebellum = 7.00, Ctrl cerebellum = 6.65, *P* = 0.85).

**Figure 1:**
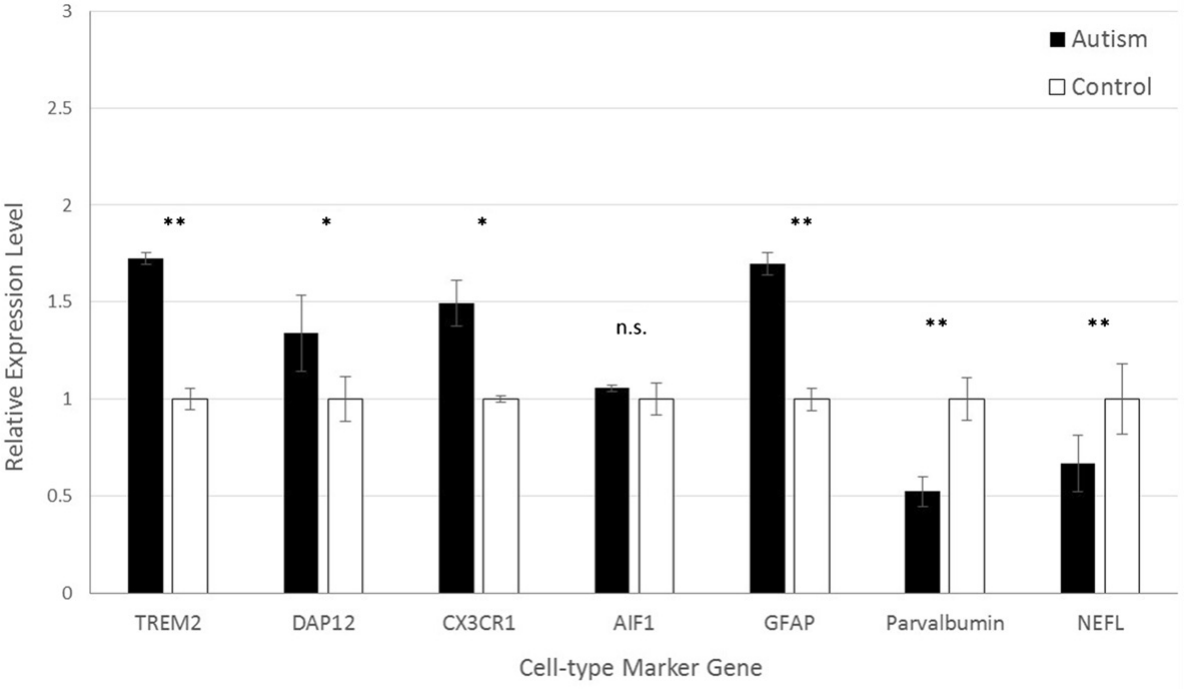
**Expression of cell-type specific markers in prefrontal cortex samples of autistic cases relative to controls.** Error bars represent 95% confidence intervals. *P <0.05, **P <0.005, n.s. = not significant

In the PFC, quantification of microglial markers demonstrated significantly increased expression in autistic samples of *TREM2*, *DAP12*, and *CX3CR1*, but not *AIF1* (Figure 1). The expression of *TREM2* was highest of all microglial markers, approximately 1.75-fold higher in autism brain tissue than controls (*P* = 0.0016). The levels of *CX3CR1* and *DAP12* were 1.50-fold (*P* = 0.0092) and 1.34-fold (*P* = 0.0086) higher in autistic samples relative to controls, respectively. Similarly, the expression of astrocyte marker *GFAP* was significantly higher in autistic brains (1.70-fold, *P* = 0.0049). Conversely, however, both the pan-neuronal marker *NEFL*, and the GABAergic interneuron-specific marker *PVA*, were significantly lower in autistic samples compared to controls (0.68-fold, *P* = 0.0034; and 0.52-fold, *P* = 0.0020, respectively).

**Figure 2:**
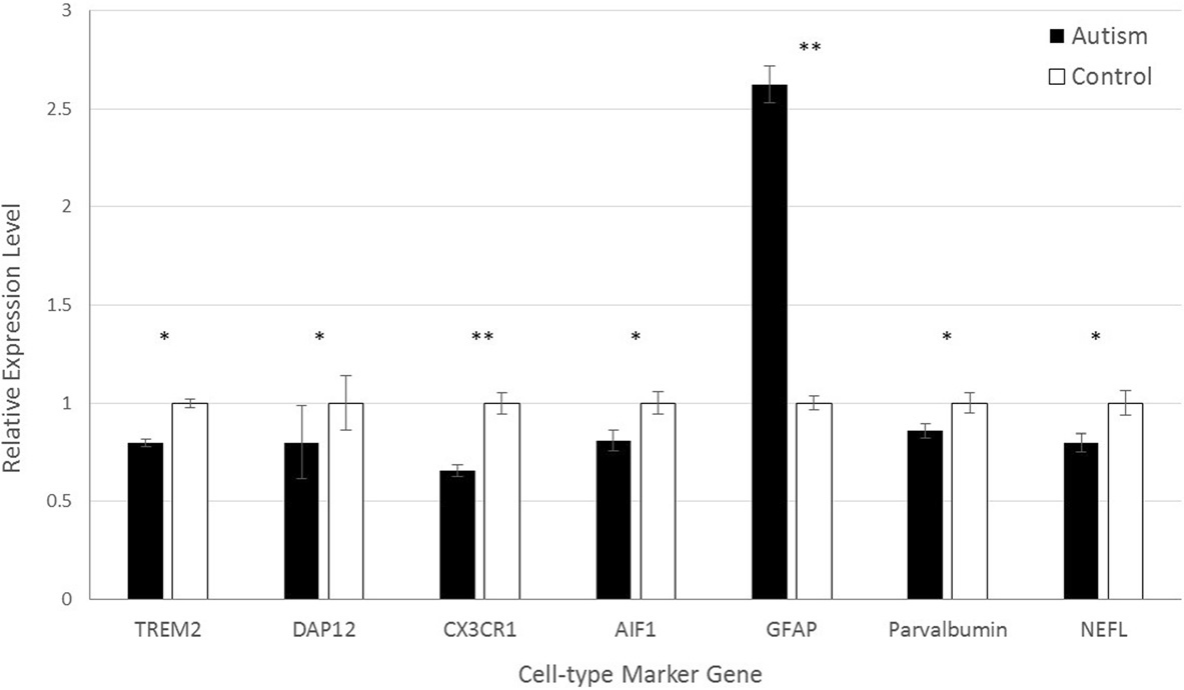
**Expression of cell-type specific markers in cerebellum samples of autistic cases relative to controls.** Error bars represent 95% confidence intervals. *P <0.05, **P <0.005.

In post-mortem cerebellum, the expression of astrocyte marker *GFAP* was also significantly higher in autism samples than in healthy controls (2.63-fold, *P* = 0.0022; Figure 2). In contrast, the expression of microglial markers *TREM2*, *DAP12*, *CX3CR1*, and *AIF1* were lower in autism tissue than in control tissue, with fold changes of 0.780 (*P* = 0.0056), 0.797 (*P* = 0.0083), 0.659 (*P* = 0.0029), and 0.808 (*P* = 0.0052), respectively. Expression of neuronal markers *PVA* and *NEFL* were also lower in autism samples than in control samples (0.862-fold, *P* = 0.033; and 0.798-fold, *P* = 0.013, respectively), as was found in the PFC.

## Discussion

While there have been multiple studies assessing RNA expression levels in autistic tissue, here we report on the expression of microglial, astrocyte, and neuron-specific cell markers concurrently in two regions of autistic brains. Our results show that glial-specific markers demonstrate altered expression in autistic brains. Moreover, the expression pattern of these cell-type specific markers parallels previous findings of glial cell number and activation patterns in autistic brain assessed via cell-level techniques (discussed below). Therefore, this approach may be a useful alternative for assessing activation and/or cell numbers in post-mortem brain tissue studies of ASD patients.

Microglia cell-marker research is still a relatively new area, and thus the markers used to quantify microglial cell number and activation are still debated. To address this issue, we used four different markers that are putatively microglial-specific. Our results demonstrate that in the PFC, there is increased expression of all microglial markers assessed, although *AIF1* did not reach statistical significance. However, previous reports have shown that *AIF1* expression in the brain is low [32], potentially contributing to this result. The finding of increased microglial cell markers in autistic PFC is in agreement with a number of studies that have found increased numbers and activation of microglia in autistic brains. For instance, Morgan et al*.* showed increased microglial density in dorsolateral PFC grey matter of ASD brains via IHC and stereology [8], and they also demonstrated that microglia are more closely associated with neurons in autistic dorsolateral PFC than in controls [9]. Similarly, Tetreault et al*.* also demonstrated increased microglial density in the frontoinsular and visual cortex of autistic brains as compared to controls [10]. Additionally, a number of studies have specifically identified microglial activation in autistic frontal cortex, through both PET radiotracer imaging [13] and IHC/cytokine profiling approaches [7]. Our results provide further support to the growing body of evidence demonstrating increased microglial numbers and activation in autistic PFC, and our cell marker gene expression results are largely in concordance with these cell level studies.

In contrast, our cerebellar results show significantly lower expression of all four microglial cell specific markers in autistic brains. While other studies have identified microglial activation in the cerebellum of autistic tissue [33], no study has attempted to specifically quantify microglial cells in the cerebellum using the markers assessed here, and therefore histopathologic studies in the cerebellum are needed to confirm these findings. One report described increased microglial cell activation in the cerebellum, assessed via HLA-DR staining in the white matter and granular cell layer of the cerebellum [7], and another showed increased microglial activation throughout the brain (although most prominently in the cerebellum) using an *in vivo* PET metabolic radiotracer [13]. However, HLA-DR expression in human microglial cells has been shown to be highly variable between individuals, and its expression actually decreases upon cytokine stimulation [34]. Moreover, as we previously discussed [35], the cerebellum is anatomically and physiologically unique; thus metabolic and pathological findings in the cerebellum must be interpreted with caution. Furthermore, our tissue samples from cerebellum contained all three layers of the cerebellar cortex, as opposed to the molecular layer only. Until direct histologic assessment of these microglial markers are performed in post-mortem autistic cerebellum, our results must be interpreted cautiously. However, our results do suggest that there are significant differences in microglial cell markers between ASD PFC and cerebellum, perhaps reflecting differences in microglial activation and/or cell numbers between these areas in ASD brain.

In both the PFC and the cerebellum, there was significantly increased expression of the astrocyte-specific marker *GFAP* in autistic brains. This trend was most prominent in the cerebellum, where *GFAP* expression was over two-fold higher in ASD brains than in healthy controls. Our findings parallel those of previous studies, which have shown increased expression of GFAP protein in the cerebellum and cortex of patients of autism through IHC staining, western blotting, and mRNA expression [7,17,36,37]. While studies have not been done to quantify astrocyte numbers in the autistic cerebellum, our results and those of previous studies provide evidence for astroglial reaction in autism.

Interestingly, we also found significantly decreased expression of the pan-neuronal marker *NEFL* in both the PFC and the cerebellum of autistic brains. This result is also supported by previous studies, which have shown decreased *NEFL* mRNA expression in the anterior cingulate gyrus, motor cortex, and thalamus of autistic brains [38]. However, cell-level studies in autistic brain have produced conflicting results about neuron numbers. While a large body of evidence has suggested there is a loss of neurons in many areas of autistic brains, as recently reviewed in [39], other studies have shown that young autistic brains may have 70% more neurons in the PFC [40]. Importantly, though, is the age of the patient at time of death, as longitudinal studies have suggested that early brain overgrowth in ASDs quickly reverses to a phenotype of neuronal loss [41]. Consequently, the older age of patients in this study may bias our findings towards the neuronal loss spectrum of the disease.

Similarly, we found significant decreases in the GABAergic interneuron-specific marker *PVA* in both the PFC and cerebellum of autistic samples*.* Despite many studies demonstrating decreased GABAergic components across different areas of the autistic brain, as reviewed in [42], the one pathological analysis of parvalbumin-positive interneurons in ASD did not identify differences in the autistic cerebellum [43]. However, this study only assessed the molecular layer of the cerebellar cortex, whereas our tissue samples contained all three layers. Additionally, while parvalbumin interneurons have been shown to be unchanged in the autistic posterior cingulate cortex and fusiform gyrus [44], and increased in the autistic hippocampus [45], they have not been directly assessed in the autistic PFC.

In addition to potentially serving as markers of glial cell numbers and/or activation, many of the genes assessed have specific putative biological relevance to ASDs themselves. For instance, in brain, *Dap12* (also known as *TYROBP*) encodes a microglial-specific transmembrane signaling polypeptide [46]. The encoded protein acts as an activating signal transduction element with known roles in brain myelination and inflammation [47]. Its receptor, *TREM2*, encodes a membrane protein that functions in modulating the brain’s immune response via production of constitutive cytokines, and is critical for activating microglial phagocytosis [48]. Rare mutations within these two genes have been associated with Nasu-Hakola disease [49]. Nasu-Hakola disease is characterized neurologically by new onset psychiatric and cognitive symptoms in the fourth decade of life, evolving to memory loss and cognitive decline resembling Alzheimer’s disease [50]. Interestingly, single nucleotide polymorphisms in this receptor pathway were also recently linked to a significantly increased risk of Alzheimer’s disease, and are thought to relate to the inability of microglia to properly remove neurodegenerative debris such as beta-amyloid [51]. It is intriguing to speculate that defects in this same pathway in neurodevelopmental disorders such as ASDs may also result in defects in microglial phagocytosis, but in the developmental context it is the inability to prune overabundant synapses, as opposed to neurodegenerative debris, that results in the behavioral phenotype.

Similarly, *CX3CR1*, also known as the fractalkine receptor, encodes a protein receptor on the surface of microglia that binds to the chemokine CX3CL1 (also called fractalkine); fractalkine functions to induce microglial migration and adhesion during phagocytosis [52]. This pathway was recently demonstrated to play a critical role in the developing brain of mice allowing for migration of microglia to their synaptic targets, where phagocytosis and synaptic refinement occur. *CX3CR1* knockout mice had more synapses on cortical neurons than wild-type mice, and displayed subtle neurological deficits [25].

*AIF1*, also known as *IBA1*, encodes a protein that is consistently up-regulated in expression during microglial activation, and therefore has been used to discriminate between resting and activated microglia [53]. The *AIF1* gene is located within the highly variable locus containing parts of the major histocompatibility complex, which itself has been linked to ASDs and dysfunctional microglia phagocytosis [54].

Glial fibrillary acidic protein (GFAP) is an intermediate filament protein that is expressed by astrocytes, and as discussed above, has been previously shown to be up-regulated in autistic brains as was also shown here. Interestingly, decreased expression of GFAP has been reported in other neurodevelopmental disorders, such as Schizophrenia and bipolar disorder [55]. GFAP, like all intermediated filament proteins, is important in maintaining the cellular cytoskeleton in astrocytes. The cytoskeleton plays a number of key functional roles in addition to maintaining cell shape, including inter-cellular communication, mitosis, and cellular migration during phagocytosis. Mutations in *GFAP* are responsible for Alexander’s disease, a rare disorder characterized by severe developmental delay, increased head size, and seizures [56].

Overall, our findings demonstrate that the autistic brain by mid-childhood has molecular changes consistent with increased astrocyte expression and decreased neuronal and interneuron expression in both the PFC and cerebellum, with PFC-specific increased microglial marker expression. Furthermore, the specific glial molecules found to be abnormal in autistic brains are intimately involved in glial mobilization and phagocytosis pathways, they have previously been shown to be critical for normal neurodevelopment, and are known to be causative of other rare neurodevelopmental phenotypes. These findings support the notion that a complex interplay between glial dysfunction and neurogenesis may underlie the clinical manifestations of ASDs.

This study has a number of limitations of note. Foremost is the relatively modest sample size. Unfortunately, post-mortem human brain research in general is hampered by the lack of accessibility to tissue samples, and pediatric samples in particular are scarce [26]. Therefore, replication with a large number of samples will be important. However, we chose qRT-PCR techniques in this pilot study because of the increased sensitivity compared to whole-genome microarray or sequencing approaches, and therefore some aspects of the small sample size limitation are addressed. Secondly, due to the inter-individual heterogeneity of the brain, and in the brain-banking methodologies used in distinguishing areas of post-mortem brain tissue, it cannot be assumed that all samples will derive from the exact same anatomic site within the PFC or cerebellum; this limitation is largely unavoidable. Lastly, the approach of using cell-type specific marker expression as a proxy for cell number and/or activation still needs verification, by assessing them concurrently with traditional histopathologic/stereology analysis. However, the concordance of our results with previously published studies, and the scarcity of appropriate ASD brain tissue and technical expertise, suggest this may be a valuable and simple alternative 'screening’ approach.

## Conclusions

In summary, assessment of glial numbers and activation in autism post-mortem brain research is hampered by the scarcity of appropriately-preserved tissue, and the technical challenge of traditional stereotactic methods. We show that glial and neuron cell-type specific markers have mRNA expression patterns that parallel known cellular aberrations in ASDs. Our results provide further evidence that glial cells may play a role in the pathogenesis of ASDs, and suggest that assessing for glial cell-type specific marker expression may represent a viable approach to relatively quantify glial cell patterns in ASD post-mortem research.

## Abbreviations

AIF1: Allograft inflammatory factor 1; ASD: Autism spectrum disorder; CX3CR1: CX3C chemokine receptor 1; IHC: Immunocytochemistry; PET: Positron emission tomography; PFC: Prefrontal cortex; PMI: Post-mortem interval; qRT-PCR: Quantitative real-time polymerase chain reaction; RIN: RNA integrity number; TREM2: Triggering receptor expressed on myeloid cells 2.

## Competing interests

The authors declare that they have no competing interests.

## Authors’ contributions

CE, MNZ, and OMR conceived of and designed the analysis. CE performed the experiments. CE, MNZ, and OMR analyzed results and wrote the manuscript. All authors read and approved the final manuscript.
